# Asymmetry in Signal Propagation between the Soma and Dendrites Plays a Key Role in Determining Dendritic Excitability in Motoneurons

**DOI:** 10.1371/journal.pone.0095454

**Published:** 2014-08-01

**Authors:** Hojeong Kim, Kelvin E. Jones, C. J. Heckman

**Affiliations:** 1 Division of Robotics Research, Daegu Gyeongbuk Institute of Science & Technology, Daegu, Korea; 2 Centre for Neuroscience and Faculty of Physical Education and Recreation, University of Alberta, Edmonton, Canada; 3 Department of Physiology, Northwestern University Feinberg School of Medicine, Chicago, United States of America; 4 Department of Physical Medicine and Rehabilitation, Northwestern University Feinberg School of Medicine, Chicago, United States of America; 5 Department of Physical Therapy and Human Movement Science, Northwestern University Feinberg School of Medicine, Chicago, United States of America; Neuroscience Campus Amsterdam, VU University, Netherlands

## Abstract

It is widely recognized that propagation of electrophysiological signals between the soma and dendrites of neurons differs depending on direction, i.e. it is asymmetric. How this asymmetry influences the activation of voltage-gated dendritic channels, and consequent neuronal behavior, remains unclear. Based on the analysis of asymmetry in several types of motoneurons, we extended our previous methodology for reducing a fully reconstructed motoneuron model to a two-compartment representation that preserved asymmetric signal propagation. The reduced models accurately replicated the dendritic excitability and the dynamics of the anatomical model involving a persistent inward current (PIC) dispersed over the dendrites. The relationship between asymmetric signal propagation and dendritic excitability was investigated using the reduced models while varying the asymmetry in signal propagation between the soma and the dendrite with PIC density constant. We found that increases in signal attenuation from soma to dendrites *increased* the activation threshold of a PIC (hypo-excitability), whereas increases in signal attenuation from dendrites to soma *decreased* the activation threshold of a PIC (hyper-excitability). These effects were so strong that reversing the asymmetry in the soma-to-dendrite vs. dendrite-to-soma attenuation, reversed the correlation between PIC threshold and distance of this current source from the soma. We propose the tight relation of the asymmetric signal propagation to the input resistance in the dendrites as a mechanism underlying the influence of the asymmetric signal propagation on the dendritic excitability. All these results emphasize the importance of maintaining the physiological asymmetry in dendritic signaling not only for normal function of the cells but also for biophysically realistic simulations of dendritic excitability.

## Introduction

Many neurons in the central nervous system have voltage gated ion channels (VGICs) in their dendrites. The activation of dendritic VGICs is location sensitive, leading to functional impacts on dendritic signaling and firing patterns [1]–[4]. Therefore, an accurate understanding of the influence of the dendritic structure on the activation of dendritic VGICs is needed. In other words, dendritic structure influences not only the passive excitability of dendrites but also their “active” excitability (here we use the phrase “dendritic excitability” to refer primarily to the active component involving dendritic VGICs).

The activation properties of dendritic VGICs were initially investigated using voltage or current clamp at the cell body [5]. Since a command signal (voltage or current) applied to the soma attenuates along the path of the dendritic trees, due to their cable properties [6], higher command signals are required to activate more distal VGICs. This effect gives rise to space-clamp as a potential confounding factor for interpretation of dendritic excitability [7], [8]. Many studies using neuron models accounted for this confounding factor by characterizing the degree of signal attenuation from the soma to the dendrites, e.g. using a coupling conductance, as the key determinant for the location dependence of VGIC activation [9]–[11].

However, studies focused on dendritic anatomy have demonstrated a more complex signal attenuation process exists between the soma and dendrites. Both experimental evidence [12] and computational analyses [13], [14] have emphasized that the attenuation of electrical signals in dendrites is asymmetric with respect to propagation *direction* (i.e. soma to dendrites and vice versa), and is moderated by *frequency* (direct (DC) or alternating (AC) current). This direction and frequency dependent dendritic signaling has been analyzed using two-port circuit theory [15] and morphoelectrotonic transformation of the anatomy of dendrites [16]. These studies suggested that a theoretical examination and understanding of dendritic excitability should go beyond the previous qualitative, or phenomenological, representation of DC signal attenuation from the soma to the dendrites. Furthermore, the previous analysis of dendritic signaling between the soma and a single point in the dendrites (i.e. point-to-point) should be extended to reflect more natural conditions in which the VGICs or synaptic inputs are positioned on many dendrites.

To systematically investigate the relationship between the complex signaling properties and dendritic excitability, the independent modulation of individual signaling properties is necessary which has been known to be very difficult in the anatomically reconstructed models. Thus, a reduced modeling approach has been developed in the ‘point-to-point’ condition between the soma and dendrites for theoretical purposes in our previous studies [17], [18]. However, it has not been validated whether the theoretical reduced models would be appropriate for the ‘point-to-all’ condition between the soma and dendrites where the VGICs or synaptic inputs are distributed in all dendritic branches over the limited range of the distance from the soma and may reproduce the input-output properties of the corresponding full model with realistic membrane excitability (i.e. Hodgkin-Huxley type) in both passive and active states.

Here we first compare the dependency of dendritic signaling on the direction and frequency among different types of spinal motoneurons in the point-to-all condition. Based on the consistent characteristics of this asymmetry across motoneuron types, we then chose one of these types in the motoneurons to provide the basis for validation of the reduced modeling approach that allows us to systematically examine the influence of the direction and frequency dependent dendritic signaling, to identify the relationship between asymmetric signal propagation and dendritic excitability. Specifically we elucidate whether the complex signaling properties of the dendrites are essential to physiologically represent dendritic excitability and how changes in these signaling properties affect normal patterns of activation of voltage sensitive channels in the dendritic tree.

## Methods and Materials

### Preparation of motoneuron anatomy

The morphological data of six type-identified cat spinal α-motoneurons (Vemoto1-6 in Burke's Lab.) was downloaded from http://NeuroMorpho.Org [19] and translated into the NEURON simulation environment v 6.1.1 using Import3D tool [20]. For individual imported motoneurons, the initial segment/axonal hillock was added to the soma after correcting the size of the cell body to the dimension previously reported [21], [22].

### Assignment of passive membrane properties

The values for the specific membrane resistivity (R_m_) were assigned to the five motoneurons (Vemoto1-4 and 6) as previously reported and used in computer simulations [21], [23], [24]. The electrotonic properties for the five motoneurons were fully addressed in our previous study [21], assuming uniform cytoplasmic resistivity (R_a_ = 70 Ω·cm) and specific membrane capacitance (C_m_ = 1 µF/cm^2^) [23]. Our initial simulations with the five anatomical motoneuron models (Vemoto1–4 and 6) used non-uniform R_m_ much lower at the soma compared to the dendrites considering the impalement resulting from sharp electrodes at the soma (i.e. somatic shunt effect) [25]–[27]. To approximate the presumed non-impaled in vivo condition, in later simulations for all six anatomical models we used a uniform R_m_ chosen to give a desired value of input resistant at the soma.

### Generation of voltage attenuation curve and voltage decay constant

**Figure 1:**
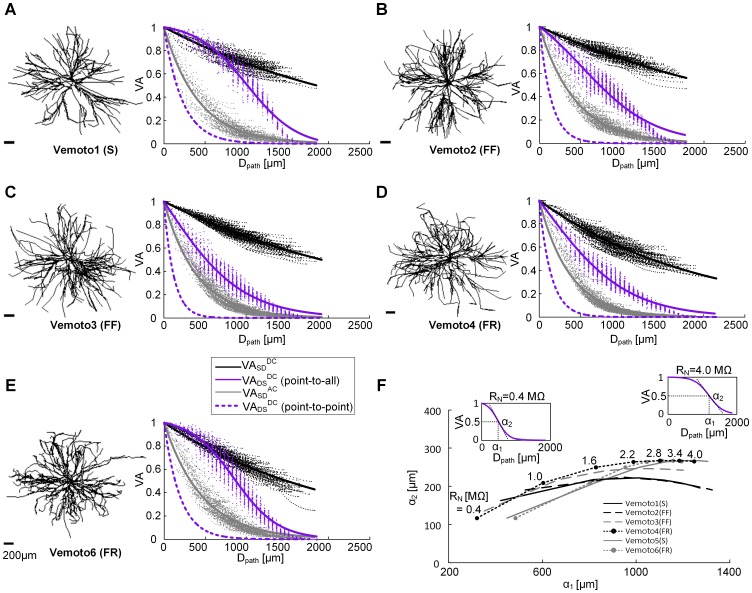
Characterization of voltage attenuation property of the dendrites. (**A**) – (**E**). Morphologies of the motoneurons in the left column and the three voltage attenuation (VA) factors measured at all distances (D_path_) from the soma (VA_SD_^DC^ in black, VA_SD_^AC^ in gray and VA_DS_^DC^ in purple) in the right column. A single exponential function (exp(-D_path_/λ)) was used to fit the VA_SD_^DC^ (λ = 2678.7, 3085.6, 2763.7, 1945.5 and 2156.4 for **A–E**) and VA_SD_^AC^ (λ = 420.1, 437.1, 402.3, 373.1 and 464.7 for **A**–**E**), and a modified Boltzmann equation (1/[1−exp(−α_1_/α_2_)+exp((D_path_−α_1_)/α_2_)]) for VA_DS_^DC^ (α_1_& α_2_ = 1020.8&307.7, 635.2&439.5, 327.9&469.6, 374.2&504.8 and 861.9&268.3 for **A**–**E**). Note that purple solid lines represents the VA_DS_^DC^ between the soma and all points in the dendrites at the same distance (i.e. point-to-all) whereas purple dotted lines (exp(-D_path_/λ) where λ = 225, 143.6, 118.5, 144, 193.7 for **A**–**E**) for the VA_DS_^DC^ between the soma and a single point along the path of all individual dendritic trees from the soma (i.e. point-to-point). (**F**). The VA_DS_^DC^ was measured in six type identified anatomically reconstructed motoneurons while varying the input resistance (R_N_) from 0.4 to 4.0 MΩ. The α_1_ (the distance at which the VA_DS_^DC^ dropped to 50%) parameter was more sensitive to changes in R_N_ compared with α_2_ parameter (a measure of the slope of the curve at α_1_). The insets show examples of the VA_DS_^DC^ curves at extreme R_N_ values of 0.4 and 4.0 MΩ. Note that the distribution of points (α_1_, α_2_) at the same R_N_ is not type specific (e.g. gray and black filled circles for FR-type motoneurons). S, FR and FF indicate slow twitch, fast twitch and fatigue resistant, and fast twitch and fast fatigable motoneuron type respectively.



The voltage attenuation (VA) factor representing signal propagation of the dendrites was defined as a ratio of voltage at the measurement site to voltage at the current injection site in the passive membrane condition [14], [15]. Both direct (DC) and alternating current (AC) were taken into account for physiological current components flowing between the soma and the dendrites: DC for steady current stimulation at the soma, tonic synaptic inputs, persistent currents at the dendrites, and AC for action potentials at the soma. Three VA factors, two for the somatofugal direction with somatically injected DC (VA_SD_^DC^) and 250 Hz-AC (VA_SD_^AC^) input and one (VA_DS_^DC^) for the somatocentric direction with simultaneous DC inputs to all points in the dendrites at the same distance from the soma, were calculated in the passive dendrites of the anatomically reconstructed neurons using the Impedance class tools in NEURON software [20]. The VA_DS_^DC^ was measured specifically for the propagation of steady synaptic inputs and persistent inward current generated by VGICs that are distributed over all branches of the dendrites at the same distance from the soma. The amount of current injection for the VA_DS_^DC^ was differentially supplied to individual points in the dendrites in proportion to the surface area of dendritic segment where each point was involved. The AC frequency of 250 Hz was calculated from the average spike width of 2 ms (or period  = 4 ms) for motoneurons [28]. The VA_SD_^AC^ for the AC signals with lower frequencies (<250 Hz) has been shown to be well conserved in our reduced modeling framework [18]. The individual VA data was plotted as a function of path length (i.e. D_path_) from the soma. To quantify the rate of attenuation with the distance, the VA_SD_^DC^ and VA_SD_^AC^ were fitted by single exponential function with voltage decay constant (λ_SD_^DC^ and λ_SD_^AC^) [18], [21]. A Boltzmann equation was arbitrarily chosen and modified to best fit the inverse-sigmoid shape of VA_DS_^DC^ data in a wide range (0.4–4.0 MΩ) of the input resistance at the soma. Using the modified Boltzmann equation, the overall profile of the VA_DS_^DC^ was characterized with two parameters (α_1_ and α_2_) where α_1_ approximately represents the distance at the VA_DS_^DC^ = 0.5 and α_2_ indicates the variation in the slope of the VA_DS_^DC^ curve at α_1_ (see Figure 1 for detailed shape). The α_1_ and α_2_ were also used in analyzing the dependency of the VA_DS_^DC^ on the motoneuron types over the full range of somatic input resistance (0.4–4.0 MΩ). The parameter values of individual fitting equations were determined using the nonlinear regression function (i.e. nlinfit) in MATLAB.

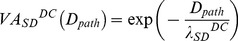
(1)

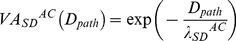
(2)

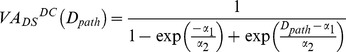
(3)


The four voltage decay constants (λ_SD_^DC^, λ_SD_^AC^ and α_1_ & α_2_ in equation (1) – (3)) specified for Vemoto6 were used to determine the values of three VA factors for the reduced models. The default values of the VA factors for the reduced model were specified at the D_path_ of 600 µm, which is an average of the distance interval (300–850 µm) previously reported for the distribution of PIC channels to produce experimentally observed nonlinear firing patterns in the anatomical model [29], [30].

### Reduced neuron model

A conductance-based, two-compartmental neuron model is used consisting of a somatic compartment and a dendritic compartment that are coupled with a single conductance. The passive dynamics of the reduced neuron model is determined by the five cable parameters: two conductances (G_m,S_ and G_m,D_) for the soma and dendrite, two capacitances (C_m,S_ and C_m,D_) for the soma and dendrite, and one coupling conductance (G_C_) between the soma and dendrite. The unique feature of our reduce modeling approach is that all five cable parameters of the reduced model are analytically determined from the five system properties (somatic input resistance (R_N_), membrane time constant (τ_m_), VA_SD_^DC^(D_path_), VA_SD_^AC^(D_path_) and VA_DS_^DC^(D_path_)) empirically measurable from real cells (see [18], [21] for details of model equations and the derivation of inverse equations for the five system properties). The five passive parameters of the reduced model were uniquely determined for the VA factors specified at the distance from the soma by solving the inverse equations,
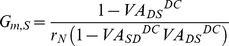
(4)

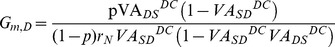
(5)

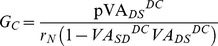
(6)

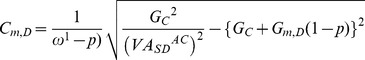
(7)

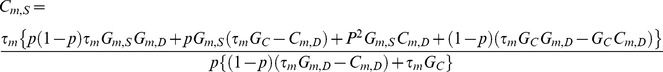
(8)


where *r_N_* is the input resistance (R_N_) normalized with the surface area of the somatic compartment; *ω* is the maximum frequency component in an action potential; *p* is the ratio of somatic to total surface area of the reduced model. In the present study, the somatic surface area (315759.2 µm^2^) of the reduced model represents total accumulated surface area from the soma up to D_path_ = 600 µm in the anatomically reconstructed motoneuron (i.e. Vemoto6). The *r_N_* and *p* were calculated from the R_N_ (1.29 MΩ) and total surface area (641786.9 µm^2^) of the Vemoto6. The default values of *r_N_* (0.407 Ω•m^2^), *τ_m_* (7.2 ms), *p* (0.492), and *ω* (2π×250 Hz calculated from the period of 4 ms assuming the spike width of 2 ms) were set to be constant at all distances (i.e. D_path_) for all reduced models used in the present study. The system equations and derivations of forward/inverse equations for the reduced model have been fully addressed in our previous studies.

### Assignment of active membrane properties

A fast twitch and fatigue resistant (FR)-type motoneuron (Vemoto6) of six anatomical models was chosen for the comparison with the reduced model. The input-output properties of the Vemoto6 has been fully analyzed in the previous study with realistic types, kinetics and distribution of voltage-gated ion channels including slowly activated, long lasting L-type Ca PIC channels in many dendrites over the limited range (i.e. 300–850 µm) of the distance from the soma [29]. The same types and kinetics of voltage gated ion channels and excitatory synapse were used for both the anatomical and reduced motoneuron model (see Appendix in [29] for details of equations and parameter values). Briefly, action potentials were generated by various active currents: fast inactivating sodium current (I_Na,f_), persistent sodium current (I_Na,p_), delayed-rectifier potassium current (I_K,Dr_), N-type calcium current (I_Ca,N_), calcium dependent potassium current (I_K(Ca)_) at the soma and I_Na,f_, I_Na,p_, I_K,Dr_ at the initial segment/axonal hillock in the anatomical model, and I_Na,f_, I_Na,p_, I_K,Dr_, I_Ca,N_, I_K(Ca)_ at the somatic compartment of the reduced model. Plateau potentials were produced by currents (I_Ca,L_) mediated by L-type Ca^2+^ channels in the dendrites of both models. In the absence of the I_Ca,L_, the active channel densities (G_Na,f_ [mS/cm^2^], G_Na,p_ [mS/cm^2^], G_K,Dr_ [mS/cm^2^], G_Ca,N_ [mS/cm^2^] and G_K(Ca)_ [mS/cm^2^]) responsible for spike generation were adjusted in both models to match the same height (i.e. 92.3 mV) of an action potential and afterhyperpolarization (AHP) properties (i.e. duration of 98.5 ms and depth of 3.1 mV) at the same rheobase current (i.e. 10.5 nA) that have been experimentally investigated without monoaminergic inputs to the motoneurons [31]. G_Ca,L_ [mS/cm^2^] for I_Ca,L_ in the dendrite of the reduced model was determined to fit the peak current (i.e. −22 nA) measured in the anatomical model during triangular voltage clamp simulations while blocking all active currents at the soma [32].

### Stimulation protocols

The voltage attenuation properties of the dendrites were characterized in the passive membrane condition with DC input (amplitude of 1 nA) for the VA_SD_^DC^ and the VA_DS_^DC^, and AC input (amplitude of 2 nA with frequency of 250 Hz) for the VA_SD_^AC^. The somatic input-somatic output relation was evaluated including the active membrane properties while applying slowly ascending and descending voltage (duration of 20 sec with peak of 30 mV starting from -70 mV) and current (duration of 10 sec with peak of 20 nA starting from 0 nA) clamp to the soma. To investigate the synaptic input-somatic output relation over a wide input range, the maximum conductance (i.e. G_syn_) of excitatory synaptic receptors in the dendrites was varied in a slowly increasing and decreasing triangular form (duration of 20 sec with peak of 1.2 mS/cm^2^ for the anatomical and 0.12 mS/cm^2^ for the reduced case starting from 0 mS/cm^2^). The peak of the G_syn_ in the reduced case was adjusted to match the same onset timing (5 ms) and peak value (−13.6 mV) of the plateau potential as the anatomical case. The smaller G_syn_ in the reduced case resulted from the larger surface area of the dendrite for Ca PIC channels compared to the anatomical case.

## Results

### Three voltage attenuation parameters to characterize dendritic signaling in neurons

Signal propagation of the dendrites was characterized in fully reconstructed motoneuron (MN) models by three voltage attenuation (VA) factors: two for the soma-to-dendrite direction with DC (VA_SD_^DC^) and AC (VA_SD_^AC^) input to the soma, and one (VA_DS_^DC^) for the dendrite-to-soma direction with simultaneous DC inputs to all points in the dendrites at the same distance from the soma. The three VA factors define neuron voltage profiles in response to steady current injected at the soma (VA_SD_^DC^), action potentials propagating from the initial segment and soma into the dendrites (VA_SD_^AC^), and steady synaptic inputs and plateau potential generated by VGICs in the dendrites (VA_DS_^DC^).

Figure 1(**A**–**E**) shows voltage attenuation as a function of distance from the soma (D_path_) in the five (i.e. Vemoto1–4 and 6) type-identified anatomically reconstructed spinal MNs. Each point in this figure shows the voltage attenuation at a single point in a dendritic branch at the indicated path distance. In all five anatomical MN models, the spatial profile of the individual VA factors was distinguishably different indicating the direction and frequency dependency of the dendritic signaling. The different dependencies of individual VA factors on the distance stem from the unbalance in electrical load between the soma and dendrites due to the highly branching structure of the dendrites and the low-pass filtering effect due to the cable properties. The vertical alignment of the data for the VA_DS_^DC^ results from spatial sampling at a resolution of 50 µm. The VA_DS_^DC^ data were fit to an inverse sigmoid curve (solid purple lines in Figure 1(**A**) and 1(**E**)), rather than the single-exponential curve (dashed purple line) previously fit to VA_DS_^DC^ when this parameter was computed between the soma and a *single* point in the dendrites [21]. The difference in DC voltage attenuation from the dendrites to the soma when calculated with the new approach was obvious in all five anatomical MN models and resulted from the summation of multiple inputs at the soma originating from the separate dendritic branches. The data from the other two voltage attenuation parameters were well fit by single exponential curves, as previously reported [18], [21]. The equations fitting individual VA data were used to calculate the values of three VA factors for the reduced MN models.

The spatial profile of the two VA factors from the soma to the dendrites was consistent across different types (slow twitch (S), fast twitch and fatigue-resistible (FR), fast twitch and fatigable (FF)) of MN models whereas the VA factor from the dendrites to the soma appeared to be related to the MN types. The S- or FR-type MN model with high input resistance (e.g. Vemoto1 (1.9 MΩ) or Vemoto6 (1.25 MΩ)) tended to be much more slowly attenuated with the distance showing the inverse sigmoid profile compared to the FF- or FR-type MN models with low input resistance (e.g. Vemoto2–3 (0.7–0.8 MΩ) or Vemoto4 (0.97 MΩ)).

Thus, we further analyzed six type-identified anatomically reconstructed MNs to evaluate how VA_DS_^DC^ generalized to a population of MNs with widely varying morphological and electrical characteristics (Figure 1(**F)**). We changed the specific membrane resistivity so that all anatomical models had the same input resistance (R_N_) at the soma, and recalculated and fit the VA_DS_^DC^ data to the inverse sigmoid curve to find the coefficients α_1_ and α_2_. The α_1_ parameter was more sensitive to changes in R_N_ compared with α_2_ parameter. All six MN morphologies had similar relationships between R_N_ and changes in α_1_ & α_2_ suggesting that the differences in morphology of the 6 models did not play a major role in VA_DS_^DC^ behavior. The evaluation of VA_SD_^DC^ and VA_SD_^AC^ for a population of MNs was presented in our previous studies [18], [21].

All these results suggest that the voltage attenuation properties in the motoneurons might not be specific to the type-related morphology of motoneurons, but a generic property of the branched architecture of dendritic trees.

### Reduced motoneuron model incorporating VA factors for dendritic excitability

One (FR-type, Vemoto6) of the anatomical MN models was chosen for our evaluation of the relationship between the signal attenuation properties and dendritic excitability since the anatomical model has been fully analyzed in the previous studies [29], [33] for the influence of dendritic PICs on MN firing patterns and synaptic integration that have been observed in various species including turtle [34], rat [35], mouse [36] and cat [37].

**Figure 2:**
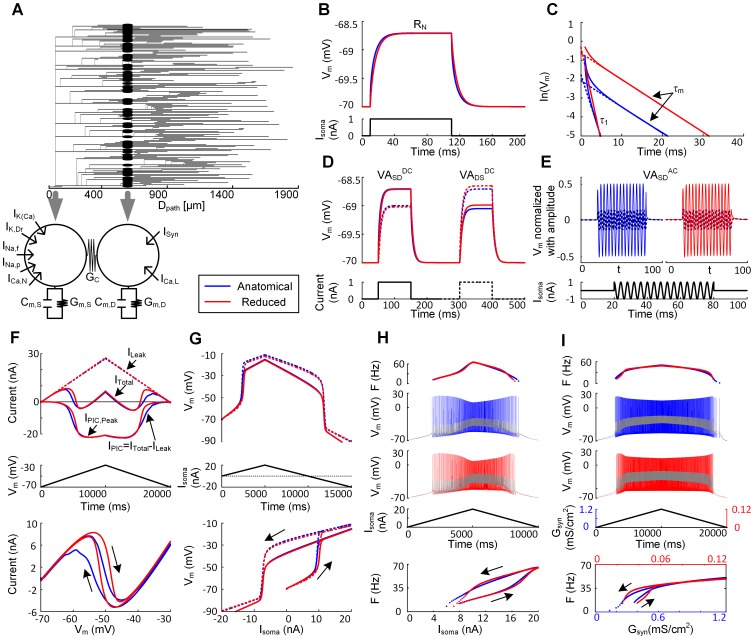
Comparison of the anatomical (blue) and reduced (red) MN model. (**A**). Reduction of anatomical dendrites. A dendrogram (upper panel) of a MN (i.e. vemoto6 in Methods) with path length (D_path_) from the soma and two compartments (bottom panel) of the reduced model representing the soma/initial segment/axonal hillock (indicated by left arrow) and all points in the narrow band (e.g. mean of 600 µm and standard deviation of 4 µm, right arrow) of dendrites of its original cell. Note that membrane potentials at individual points in the dendrites of the anatomical model were averaged for the purpose of comparison to the reduced model. (**B**) – (**E**). Passive dynamics. All five passive parameters (G_m,S_ = 0.143 mS/cm^2^, G_m,D_ = 0.131 mS/cm^2^, C_m,S_ = 1.058 µF/cm^2^, C_m,D_ = 0.915 µF/cm^2^, G_C_ = 0.211 mS/cm^2^) of the reduced model were analytically determined to retain the five system properties measured from the anatomical model: input resistance (R_N_ = 1.29 MΩ) and membrane time constant (τ_m_ = 7.2 ms) obtained by peeling analysis at the soma, and three voltage attenuation factors (VA_SD_^DC^ = 0.76, VA_DS_^DC^ = 0.75, VA_SD_^AC^ = 0.27) at D_path_ = 600 µm. (**B**). Time course of membrane potential (V_m_) in response to the step current (I_soma_) injected to the soma for calculating R_N_. (**C**). The semilog plot (solid lines) of transient voltage response at the soma to brief current pulse with smaller amplitude for anatomical than reduced case for τ_m_ and τ_1_ (equalizing time constant) equivalent to the inverse of slope of linear regression (dotted) line fitting the tail of each curve. (**D**). Time courses of V_m_ for VA_SD_^DC^ from the soma (solid lines) to dendrites (dotted lines) and VA_DS_^DC^ in the opposite direction, in response to DC input (bottom panel) to the soma (solid line) and dendrites (dotted line). (**E**). Time course of V_m_ normalized with its amplitude for VA_SD_^AC^ from the soma (solid line) to dendrites (dotted line), in response to somatically injected AC current (I_soma_) with the frequency of 250 Hz. (**F**), (**G**). PIC activation with the addition of I_Ca,L_ to the dendrites of both models. (**F**). Time course of effective PIC (I_PIC_, upper panel) obtained by subtracting leak (I_Leak_) from total current (I_Total_) injected for following the triangular voltage-clamp (V_m_, middle panel) at the soma, and I_Total_-V_m_ relationship (bottom panel). (**G**). Time course of V_m_ (upper panel) at the soma (solid lines) and dendrites (dotted lines) in response to triangular current clamp at the soma (I_soma_, middle panel), and V_m_-I_soma_ relationship (bottom panel). Arrows indicate the ascending and descending phase of triangular voltage and current clamp. Maximum conductance (G_Ca,L_) for I_Ca,L_ was 1.37 mS/cm^2^ for the anatomical and 0.124 mS/cm^2^ for the reduced case. (**H**), (**I**). Cellular excitability with the addition of I_Na,f_, I_Na,p_, I_K,Dr_, I_Ca,N_ and I_K(Ca)_ to the soma of both models. (**H**). Instantaneous firing rates (F, upper panel), time course of V_m_ (blue & red for the soma, and gray for the dendrite) in response to triangular current stimulation (I_soma_) to the soma, and F-I_soma_ curve (bottom panel). Maximum conductances for the active currents in the anatomical case were G_Na,f_ = 0.71 [S/cm^2^], G_K,Dr_ = 0.23 [S/cm^2^], G_Ca,N_ = 0.01 [S/cm^2^], G_K(Ca)_ = 0.0258 [S/cm^2^] at the soma, G_Na,f_ = 2.7 [S/cm^2^], G_Na,p_ = 0.033*10^−3^ [S/cm^2^], G_K,Dr_ = 0.17 [S/cm^2^] at the initial segment and axon hillock, and G_Ca,L_ = 1.37 [mS/cm^2^] in the dendrites. For the reduced model, G_Na,f_ = 26.75 [mS/cm^2^], G_Na,p_ = 0.00086 [mS/cm^2^], G_K,Dr_ = 6.2 [mS/cm^2^], G_Ca,N_ = 0.008 [mS/cm^2^], G_K(Ca)_ = 0.54 [mS/cm^2^] at the soma and G_Ca,L_ = 0.124 [mS/cm^2^] at the dendrite. (**I**). F, V_m_ (blue & red for the soma, and gray for the dendrite) in response to triangular variation of the maximum conductance (G_syn_) for the synaptic receptors positioned at all points at D_path_ = 600 µm, and F-G_syn_ curve. Arrows indicate the ascending and descending phase of G_syn_.

To explicitly evaluate the influence of the three VA factors on the location dependence of dendritic excitability, the anatomically reconstructed MN (i.e. Vemoto6) was reduced using our recently developed two-compartment model that analytically retained the VA factors of its original cell as well as whole cell properties measured at the soma such as input resistance (R_N_) and system time constant (τ_m_) [18] (Figure 2(**A**)). For the validation of the reduced model in physiological realism, the systematic comparison between the full anatomical model and the reduced model was conducted in two steps. Firstly, all levels of input/output relations were compared at D_path_ = 600 µm with respect to: the passive membrane properties, the dendritic excitability with PIC channels in the dendrites, and the cellular excitability for firing output at the soma. Then, we constructed multiple two-compartment models and matched their excitability for D_path_ values along the dendrites of the full model.

#### Comparison of passive dynamics of the reduced and full models

The five system parameters (i.e. R_N_, τ_m_, and VA_SD_^DC^, VA_DS_^DC^ and VA_SD_^AC^ for D_path_ = 600 µm) used to solve for the passive properties of the reduced model, were sufficient to replicate the desired passive membrane features of the anatomical model (Figure 2(**B**) & (**C**)). The time course of depolarization and repolarization in response to the long lasting step current at the soma was very similar in both cases (Figure 2(**B**)), despite the slight difference in the second time constant (or equalizing time constant, τ_1_ in Figure 2(**C**)). To avoid the overlap of lines due to the same input resistance between the anatomical and reduced model, brief current pulse with smaller amplitude was applied to the soma for the anatomical (blue) than reduced (red) case resulting in the downward shift of the voltage response for the anatomical case in Figure 2(**C**). The somatic and dendritic membrane potentials measured for the calculation of VA_SD_^DC^ were identical, whereas those for the VA_DS_^DC^ were slightly larger by about 0.09% in the reduced model compared to the anatomical model at steady state (Figure 2(**D**)). The similarity of dendritic depolarization in the two models, by the same dendritically injected current, indicates that the input resistance at the dendrite of the reduced model captures the input resistance of the complex dendritic network of the anatomical model at the D_path_ = 600 µm. The amplitude ratio (i.e. VA_SD_^AC^) between somatic and dendritic membrane potential in response to 250 Hz-AC input to the soma was the same between two models (Figure 2(**E**)).

#### Comparison of dendritic PIC activation

The next comparison was for dendritic PIC activation during either slow-ramp voltage or current clamp applied to the soma, while blocking all active currents generated at the soma. In this way, we could isolate the influence of the DC signal attenuation (i.e. VA_SD_^DC^ and VA_DS_^DC^) on the PIC activation from that of the AC signal attenuation (i.e. VA_SD_^AC^). The maximum conductance of the Ca channels in the dendrite of the reduced model was fit to the peak current measured in the anatomical model during triangular voltage clamp simulations (I_PIC,Peak_ in Figure 2(**F**)). During the rising phase of triangular voltage clamp the two models produced similar N-shaped dynamics of the total current (I_Total_, top panel in Figure 2(**F**)). The consequence of PIC activation was a long lasting depolarization (plateau potential, top panel in Figure 2(**G**)) at both soma and dendrites during the triangular current stimulation to the soma. The delayed offset of the PIC during the falling phase of voltage clamp relative to the rising phase of voltage clamp resulted in hysteresis of the current/voltage relationships (bottom panels in Figure 2(**F**) & (**G**)). A difference between the two models was the earlier onset and slower offset of the PIC in the anatomical dendrites, probably due to the variance in the input resistance of individual dendritic branches at the D_path_ of 600 µm. However, this difference was negligible in current clamp conditions during activation of the plateau potential (Figure 2(**G**)).

#### Comparison of firing responses

Because the activation of PIC channels in the dendrites is influenced by action potentials propagating into the dendrites [18], we compared firing patterns in response to the triangular current stimulation to the soma between the anatomical and reduced models. In the presence of Ca PIC channels, the two models showed similar nonlinear firing behavior (bottom panel in Figure 2(**H**)), i.e. counter-clockwise hysteretic frequency-current (F-I_soma_) relationship with sustained firing below the current threshold for spike initiation. The firing rate at the end of the descending phase of the current injection is greater for the anatomical model because the slower offset of the PIC results in more inward current. Overall there was a good match of firing rate behavior in the two models. Figure 2(**I**) shows that the reduced model also matched the full model even in the case where tonic synaptic inputs interacted with PIC channels in the dendrites [38]. The amplitude of membrane potentials at the soma and dendrites was similar between the two models during the triangular changes in G_syn_. Consistent with the F-I_soma_ relationship with somatically injected current (bottom panel in Figure 2(**I**)), the anatomical model had the faster onset of the PIC channels and longer duration of sustained firing below the threshold for the spiking than the reduced model.

#### Comparison of the effects of varying the distance for PIC location

**Figure 3:**
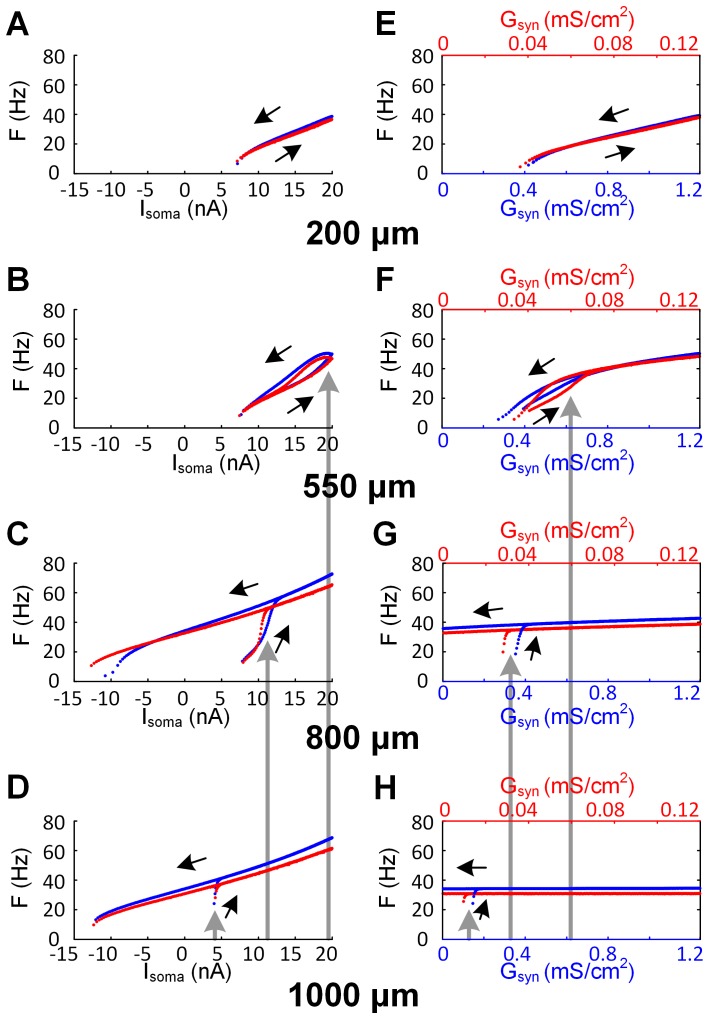
Cellular excitability of the anatomical (blue) and reduced (red) model with different locations of PIC channels in the dendrites. The neuron models and stimulation protocols were the same as those used in Figure 2(H) & (I) except for variation of PIC channel location in the dendrites. (A) – (D). Firing rates (F) in response to triangular current stimulation to the soma (I_soma_). (E) – (H). Firing rates (F) in response to triangular variation of maximum conductance (G_syn_) of excitatory synaptic receptors in the dendrites. The direction of black arrows indicates the ascending and descending phase of triangular current stimulation and change in G_syn_. The gray arrows indicate the current and G_syn_ threshold for activating plateau potentials at different distances from the soma. Note the negative relation between the distance and input threshold. At different locations in the dendrites, the three VA factors were VA_SD_^DC^ = {0.91, 0.77, 0.69, 0.63}, VA_DS_^DC^ = {0.96, 0.79, 0.57, 0.38} and VA_SD_^AC^ = {0.65, 0.31, 0.18, 0.12}; the density of Ca PIC channels were G_Ca,L_ = {1.14, 1.27, 1.95, 4.1 mS/cm^2^} in the anatomical and {0.11, 0.122, 0.132, 0.189 mS/cm^2^} in the reduced model; the passive parameter values of the reduced model were G_m,S_ = {0.078, 0.132, 0.174, 0.2 mS/cm^2^}, G_m,D_ = {0.179, 0.143, 0.1, 0.07 mS/cm^2^}, G_C_ = {0.918, 0.244, 0.114, 0.06 mS/cm^2^}, C_m,S_ = {0.609, 1.077, 1.211, 1.302 µF/cm^2^}, C_m,D_ = {1.239, 0.903, 0.764, 0.62 µF/cm^2^}. Parameter values in the parentheses are in the order of increasing distance from 200 to 1000 µm.

The above results only applied to the case where the PIC channels were localized at all points of the dendritic branches separated by about 600 microns away from the soma in both the full and reduced models (see Figure 2A for graphical description). We next varied the location of the ion channels that generate the PIC. For each location, a new two-compartment model was created and matched to the passive properties (R_N_, τ_m_ and three VAs) of the anatomical model. This required location dependent alterations in the VA parameters of the reduced models. The PIC channel density of both models was adjusted to maintain a constant peak of I_PIC_ determined at the D_path_ = 600 µm [32]. In general, the reduced model was able to predict the overall firing pattern of the anatomical model at all distances from the soma during either somatic current stimulation or synaptic input (Figure 3). As the PIC channels moved towards the dendritic terminals, both threshold current and synaptic conductance for the activation of plateau potential decreased (gray arrows in Figure 3). These results indicate that the more distant the location of the PIC channels in the dendrites, the more “excitable” the cell is.

### Dependency of dendritic excitability on the VA factors

We have quantitatively characterized the voltage attenuation properties of the dendrites using the anatomically reconstructed MN models (Figure 1) and showed that physiological changes in the VA factors for the two- compartment models allowed representation of the spatial variation in the threshold for activating plateau potentials along the path of the complex MN dendrites (Figure 2 and 3). Therefore, we next used the realistic two-compartment models to systematically evaluate how the VA factors influence dendritic excitability. To achieve this, a sensitivity analysis of the effect of the VA factors on dendritic PIC activation was conducted for PIC channels located at different distances from the soma.

#### Sensitivity of cellular excitability to the VA factors

**Figure 4:**
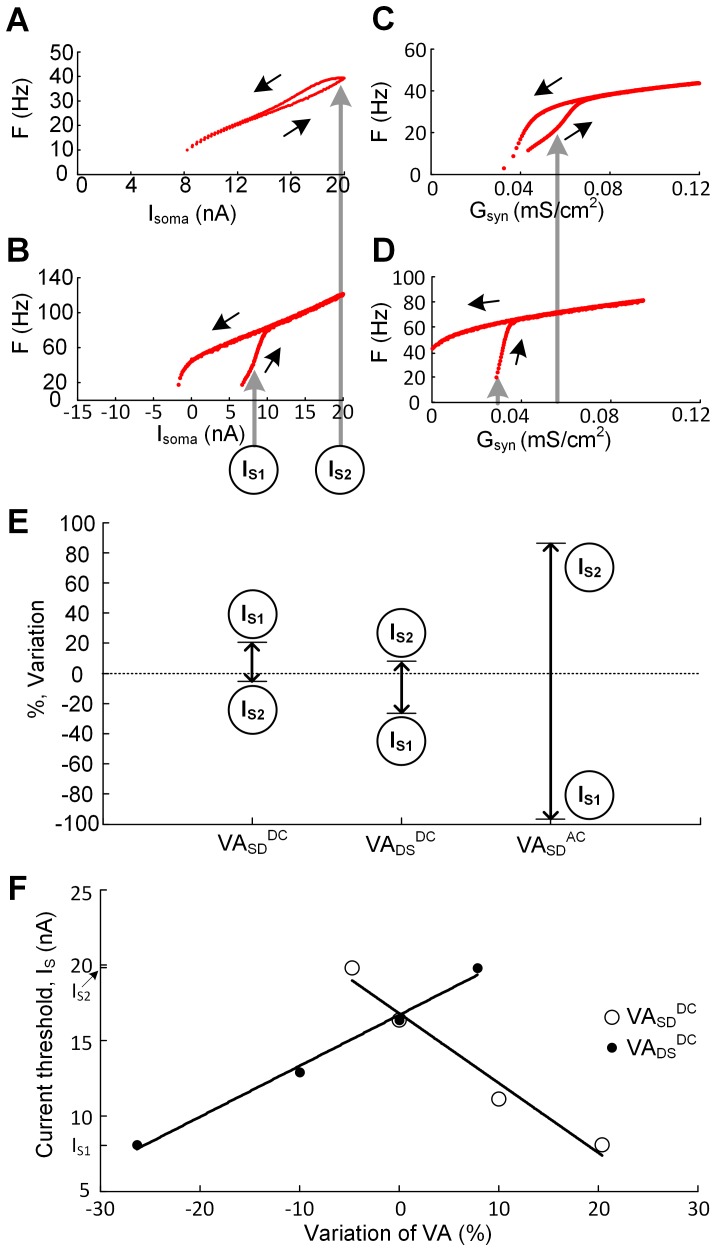
Sensitivity of cellular excitability to the VA factors. Using the same reduced model and ramp stimulation protocols used in Figure 2(**H**) & (**I**), F-I_soma_ and F-G_syn_ relationships were simulated while varying individual VA factors independently. (**A**), (**B**). F-I_soma_ curves with VA_SD_^DC^ decreased by 4.7% and increased by 20.4% from its original value (0.76). Circled letters, I_S1_ (8.1 nA) and I_S2_ (19.8 nA), on vertical gray lines indicate decreased and increased current threshold for initiating firing acceleration as a result of changing the VA_SD_^DC^. Black arrows indicate the rising and falling phase of triangular current stimulation to the soma. (**C**), (**D**). F-G_syn_ curves with VA_SD_^DC^ changed by the same variation (%) as applied to (**A**) and (**B**). Vertical gray arrows indicate increased (about 0.055 mS/cm^2^, left column) and decreased (about 0.029 mS/cm^2^, right column) threshold-G_syn_ for the firing acceleration as a result of varying the VA_SD_^DC^. Black arrows indicate the rising and falling phase of triangular change in G_syn_ at the dendrite. (**E**). The percent variation in individual VA factors (VA_SD_^DC^, VA_DS_^DC^, VA_SD_^AC^) to produce the firing acceleration at either I_S1_ or I_S2_. 0% represents the original values of VA factors and minus sign in the ordinate indicates the decrease in VA factor values. (**F**). Relationship of threshold current (I_S_) for the firing acceleration and the percent variation in DC VA factors. Data points were fitted to a linear regression line. Note that R_N_ for the reduced model was set to be 1.29 MΩ.

Triangular currents were injected at the soma to generate frequency-current (F-I_soma_) curves and the threshold for PIC activation was estimated from the acceleration in firing. When VA_SD_^DC^ was decreased by 4.7% from its original value (0.76), a high threshold was detected at 19.8 nA and labeled I_S2_ (Figure 4(**A**)). Similarly, when VA_SD_^DC^ was increased by 20.4%, a low current threshold for this acceleration was detected at 8.1 nA and labeled I_S1_ (Figure 4(**B**)). A shift from I_S2_, to I_S1_ represents a hyperpolarizing shift of the threshold, indicating an increase in excitability. The sensitivity of each of the three voltage attenuation factors was inferred by measuring the amount of variation required to alter the acceleration in firing between I_S1_ and I_S2_. We inferred high sensitivity to a VA factor when small variations were required to achieve the target thresholds. The threshold for the firing acceleration was more sensitive to changes in the DC than AC VA factors (Figure 4(**E**)). Variation of the DC VA factors changed the threshold targets in opposite directions. Increasing the attenuation from the dendrites to the soma by decreasing VA_DS_^DC^ resulted in an increase in excitability. In contrast, increasing the attenuation in the opposite direction, VA_SD_^DC^, produced a decrease in excitability. Therefore, we conclude that VA_SD_^DC^ is positively correlated, whereas VA_DS_^DC^ is negatively correlated with excitability (Figure 4(**F**)). The slope of the regression lines indicates that current threshold is most sensitive to voltage attenuation from the soma to the dendrites. The relatively modest effects of changes in VA_SD_^AC^ also increase excitability.

Similar effects of varying individual voltage attenuation factors were found in the case where the PIC was activated by synaptic conductances applied to the dendrites (Figure 4(**C**) & (**D**)). The three voltage attenuation factors showed the same correlations with excitability to synaptic input as current input to the soma. That is, increases in VA_SD_^DC^ increased excitability, whereas increases in VA_DS_^DC^ or VA_SD_^AC^ decreased excitability.

#### Role for the VA factors in the spatial profile of dendritic excitability

**Figure 5:**
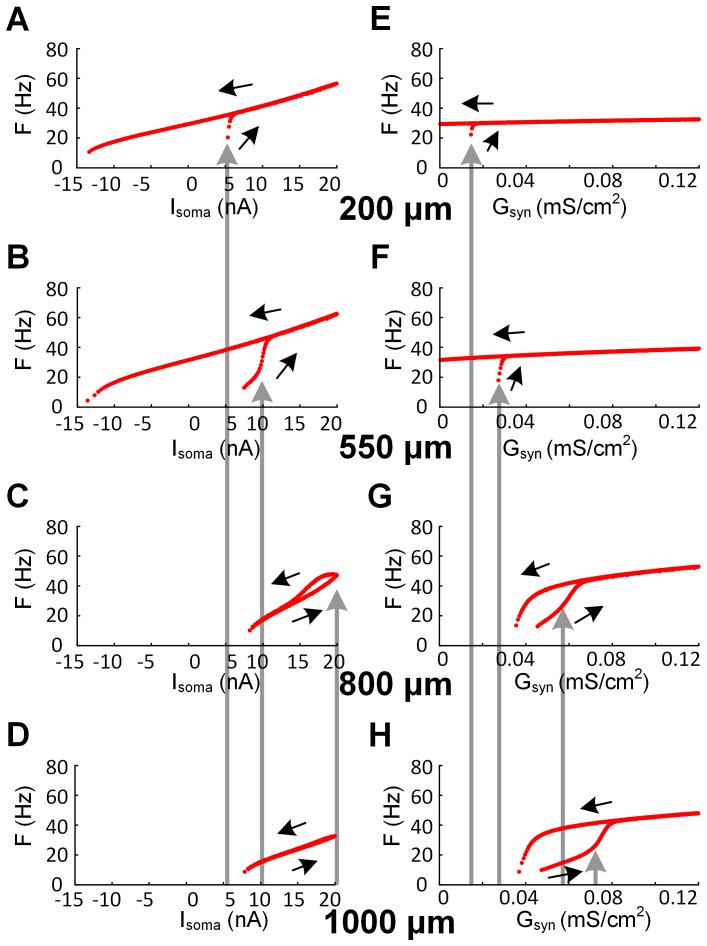
Spatial dependence of cellular excitability on VA_DS_^DC^ and VA_SD_^AC^. All simulation conditions were identical to those used in Figure 4 except for systematically varying the values of the VA_DS_^DC^ and VA_SD_^AC^. Note the inverse order of firing patterns with increasing distance compared to the physiological case (Figure 3). (**A**) – (**D**). F-I_soma_ relationships. (**E**) – (**H**). F-G_syn_ relationships. The direction of black arrows indicates the ascending and descending phase of triangular current stimulation and change in G_syn_. The gray arrows indicate the current and G_syn_ threshold for activating plateau potentials at different distances from the soma. At different locations, the VA_DS_^DC^ and VA_SD_^AC^ were {0.47, 0.6, 0.78, 0.876} and {0.65, 0.31, 0.18, 0.5}. The passive parameter values of the reduced model were G_m,S_ = {0.228, 0.183, 0.117, 0.067 mS/cm^2^}, G_m,D_ = {0.019, 0.079, 0.181, 0.276 mS/cm^2^}, G_C_ = {0.099, 0.135, 0.204, 0.234 mS/cm^2^}, C_m,S_ = {1.644, 1.387, 0.766, 1.617 µF/cm^2^}, C_m,D_ = {0.134, 0.499, 1.373, 0.352 µF/cm^2^}. Parameter values in the parentheses are in the order of increasing distance from 200 to 1000 µm. Note that R_N_ for all reduced models was set to be 1.29 MΩ.

We next explored how the voltage attenuation factors affected the relationship between PIC location along the dendrites and its threshold for activation. When all three VA factors were constrained to biophysical values obtained from simulations with the anatomical MN model (Figure 1(**A**)), moving the PIC location close to the soma resulted in an increase in PIC activation threshold: the closer the PIC, the greater the current (either injected or synaptic) required to activate it (Figure 3). In the present analysis, we held VA_SD_^DC^ at its physiological values for each PIC distance and varied VA_DS_^DC^ and VA_SD_^AC^. The results of these changes were striking in that the spatial order of the PIC activation observed in the physiological case (Figure 3) could be inverted, as illustrated in Figure 5. That is, PIC threshold activation shifted to lower values as its location was moved closer to the soma. As a result, variations in VA_DS_^DC^ and VA_SD_^AC^ could be found to match the firing patterns when all three VA factors were constrained to their physiological ranges (Figure 3) with the PIC at non-physiological locations. For instance, the F-I_soma_ curve at D_path_ = 1000 µm in the physiological case (Figure 3(**D**) & (**H**)) could be reproduced at D_path_ = 200 µm by decreasing only the VA_DS_^DC^ from 0.96 to 0.47 (Figure 5(**A**) & (**E**)), whereas the increase in both the VA_DS_^DC^ (0.63→0.876) and VA_SD_^AC^ (0.12→0.5) could lead to the same F-I_soma_ curve at D_path_ = 200 µm of the physiological case (Figure 3(**A**)) at D_path_ = 1000 µm (Figure 5(D)). The strong dependency of the PIC activation and associated firing patterns on the VA_DC_^DC^ and VA_SD_^AC^, at all positions in the dendrites, indicates that all three voltage attenuation factors are necessary for biophysically realistic simulations of dendritic excitability.

## Discussion

The high dimensionality of dendritic systems has made it challenging to get insights into whether and how the complex (directional and frequency-dependent) dendritic signaling contributes to the activation of voltage gated ion channels in dendrites. We have demonstrated using realistically reduced MN models that the asymmetry in voltage attenuation between the soma and the dendrites plays an essential role in determining the spatial activation pattern of voltage sensitive dendritic channels and associated somatic firing output. Our results also showed that voltage attenuation properties in several different types of neurons have an asymmetric profile that is remarkably similar to that in spinal MNs, suggesting that the essential role of this asymmetry for normal function may apply widely in neurons. All these results support the conclusion that the biophysically based asymmetry in signal propagation of the dendrites should be maintained in reduced models of neurons to physiologically represent the dendritic excitability.

### Interaction of voltage attenuation factors with dendritic excitability

The asymmetry of the dendritic signaling has been quantified by a ratio (VA_SD_^DC^/VA_DS_^DC^) of DC voltage attenuation factors between the soma and a single point in the dendrites [17]. This asymmetry index has been theoretically shown to be proportional to input resistance (R_N,D_) at the same site of the dendrites leading to the equation [17],
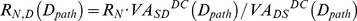
(9)where the R_N_ is assumed to be constant for a single neuron during the variation in the DC VA factors.

**Figure 6:**
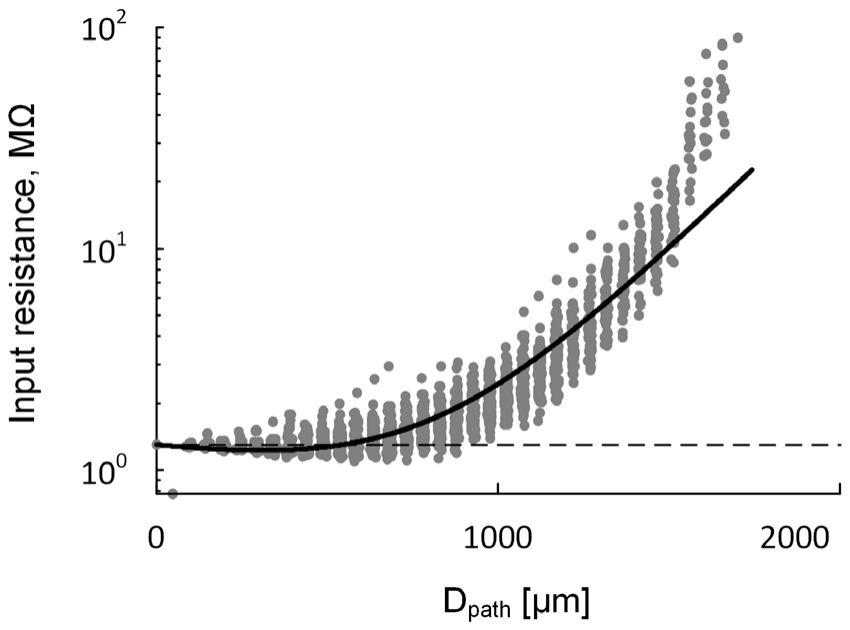
Dependence of input resistance at the dendrites on the DC VA factors. Input resistance (R_N,D,_ gray dots) was measured at individual points of the motoneuron dendrites at the same distance with respect to the total current injected to the dendrites (see the stimulation protocol used for the VA_DS_^DC^). The measured R_N,D_ was superimposed by the R_N,D_ (black line) that was predicted from fitting curves to the DC VA data using the equation (R_N,D_(D_path_) = R_N_*VA_SD_^DC^(D_path_)/VA_DS_^DC^(D_path_) where R_N_ is input resistance at the soma (D_path_ = 0)). Note the agreement between the predicted and measured input resistance in the dendrites. The poor fit at the distal distance was attributed to the error between the VA_DS_^DC^ data and its fitting curve (see Figure 1(**A**)).

In the present study, the VA_DS_^DC^ was characterized between the soma and all points of the dendrites at the same distance. Our further analysis confirmed that the input resistance predicted with the equation well matched that measured directly from the anatomical model (Figure 6). This result indicates that in the case of ‘point-to-all points’ the complex distribution of R_N,D_ in the dendrites could also be captured by the same equation as for the case of ‘point-to-point’.

It should be first noted that the activation of the PIC may be significantly influenced by both the location and density of the PIC channels in the dendrites. The sensitivity analysis of the PIC activation to individual VA factors was performed at a specific location (i.e. 600 µm) in the dendrites while keeping the density of the PIC channel constant (Figure 4). Our further analysis confirmed the same results at all distances from the soma with the PIC density constant. Under this condition, the influence of the asymmetric signal propagation on the PIC activation in the dendrites may be explained directly from the relationship between the DC VA factors and R_N,D_ in the equation (9).

The equation derived directly from the reduced modeling approach may provide crucial insights into how the asymmetric signal propagation in the dendrites influences the activation threshold for plateau potential and the firing behavior of the neurons. Figure 6 showed that the spatial variation in the input resistance (R_N,D_) of the dendrites was directly related to the spatial variation in the DC VA factors, VA_SD_^DC^ and VA_DS_^DC^. The decrease in the threshold for PIC activation with increasing distance that have been shown during both somatic and dendritic excitation may be explained by the increase in the dendritic input resistance resulting from decrease in VA_DS_^DC^ as the distance increases (Figure 3). This result was consistent for a broad range of variation in individual voltage attenuation factors at a specific PIC location (Figure 4). The prediction could also be made for Figure 5 taking into account the decreasing R_N,D_ due to the increase in the VA_DS_^DC^ while holding the VA_SD_^DC^ in a physiological range. All these results propose the tight relation of the asymmetric signal propagation to the input resistance in the dendrites as a mechanism underlying the influence of the asymmetric signal propagation on the dendritic excitability in motoneurons.

In the present study, the attenuation of AC signals has been considered only for the propagation from the soma to the dendrites assuming the tonic synaptic inputs to the dendrites. The reason for that was not only due to the limitation of the current reduced modeling approach (see Methods), but also the impact of back-propagating action potentials on the dendritic excitability (compare current threshold for the plateau potential at the dendrites between Figure 2(**G**) and 2(**H**)). The action potentials passively propagating into the dendrites may have both excitatory and inhibitory effects on the activation of PIC channels by their spike and afterhyperpolarization (AHP) phase. The contribution of the passive dendrites to the back-propagation of action potentials was characterized with the VA_SD_^AC^ which negatively related to the signal frequency. Because the duration of AHP (e.g. 100 ms) is much longer than spike (e.g. 2 ms) in MNs, the inhibitory effect of back-propagating action potentials is expected to be dominant rather than the excitatory effect (compare the current threshold for PIC activation between Figure 2(**G**) and 2(**H**)). This inhibitory effect has been also shown to be important to shape the graded activation of plateau potentials, preventing from being activated in an all-or-none manner [33]. Due to exponential decay of the VA_SD_^AC^ with increasing distance, the back-propagating action potentials would hamper the activation of PIC channels around the soma more effectively. This implies that the VGICs in proximal dendritic sites would be even harder to be activated relative to distal sites. The similar non-uniformity of dendritic excitability, lower around the soma than distal dendrites, would be expected to occur in other major types of neurons in the brain because they showed the similar profile of the three VA factors as a function of the distance from the soma (Figure 1).

Many computational studies on the nonlinear firing patterns of MNs have suggested that L-type Ca^2+^ channels mediating plateau potentials should be clustered in the dendrites away from the soma to generate the nonlinear firing patterns (hysteretic F-I_soma_ relationship, bottom panel in Figure 2(**H**)) [39], [40]. However recent immunohistochemical study has shown the high densities of CaV1.3 clustered at both proximal (<100 µm) and distal sites (450∼650 µm) from the soma [41]. Furthermore the nucleated patch clamp recordings from an isolated soma without the dendrites have revealed the existence of Ca PIC channels even at the soma [42]. However, the distal PIC channels may be predominant in determining firing patterns during the current stimulation to the soma because of the higher excitability at the distal dendritic region relative to the proximal sites (Figure 3 and 6).

The location dependent excitability in the dendrites was associated with input/output relationships of the model MN (Figure 3). While varying the location of PIC channels, the MN could reproduce all nonlinear firing patterns experimentally observed in MNs. Four firing types (i.e. Type I-IV) have been classified based on the F-I relationship during triangular current stimulation to the soma [43]. The Type I (linearly overlapping F-I relationship without self-sustained firing) or II firing (clockwise F-I relationship without self-sustained firing) was observed near the soma (<300 µm) whereas the Type III (linearly overlapping F-I relationship with self-sustained firing) or IV (counterclockwise F-I relationship with self-sustained firing) tended to occur in a dendritic region separated further away from the soma (>600 µm). A variance in firing patterns between MNs might be due to the different distribution of PIC channels in the dendrites, instead of variation in active channel properties.

### Variability in voltage attenuation properties and dendritic excitability

The distance dependent VA property of the dendrites is potentially tunable during normal behavior of neural networks. In a MN pool of cat medial gastrocnemius muscle, electrical properties including R_N_, τ_m_ and AHP properties have been reported to be systematically related to the types (S-, FR- and FF-type) of MNs in a continuous manner [44]. R_N_ has been shown to have positive effect on the reduction of voltage attenuation by altering the spatial profile of individual VA factors: VA_SD_^DC^ [21], VA_SD_^AC^ [18] and VA_DS_^DC^ (Figure 1(**F**)). The τ_m_ and AHP properties may further influence the frequency dependent VA_SD_^AC^ because they are key parameters determining the shape of action potentials. In addition, the membrane electrical properties of individual neurons could be modulated by the different levels of background activity or shunting effect by synapses bombarding the dendritic trees [45], [46]. Furthermore the properties of leak current channels determining the cable property might be targeted by a variety of diffusive neurotransmitters to manipulate the spatial dendritic excitability [47], [48]. In addition, dendritic morphology is not static, but dynamically varying in physiological conditions. Subtle changes in morphology may also occur due to a various pathological conditions [49]-[51] or physical damage [52]. All these electrical and morphological changes may influence the VA properties of the dendrites and significantly alter the spatial profile of dendritic excitability, enriching the repertoire of input/output functions of neurons within neural networks without changing physical location or activation properties of VGICs in the dendrites [53], [54].

### Limitations of the current modeling approach

The asymmetric signal propagation properties of the complex dendrites are implicitly embedded in the anatomically reconstructed neuron models. To explicitly manipulate the asymmetric properties of dendritic signaling and demonstrate their effects on dendritic excitability, we have developed the analytical, two-compartment framework consisting of the somatic and dendritic compartment. In the present study, our previous approach for the asymmetric signal propagation between the soma and a single point in the dendrites has been extended for more natural conditions of the asymmetric signal propagation between the soma and all points in the dendrites at the distance from the soma. For physiological analysis of the role of the asymmetry between the soma and dendrites for dendritic excitability, both passive and active behaviors of the extended model have been validated comparing directly to the input-output properties of its full model with realistic types, kinetics and distribution of voltage-gated ion channels at the soma and dendrites.

However, the fundamental limitations of the two-compartment modeling approach have raised several issues in the present study. The first one is that the collapse of the branching structure of the dendrites into a single compartment sacrificed the details for the activation and deactivation dynamics of PICs positioned at individual branches at the same distance from the soma. Consequently, the PIC (i.e. I_PIC_) measured at the soma of the anatomical model was more rapidly activated during the rising stimulation phase and more slowly deactivated during the falling stimulation phase compared to the reduced case. In turn, this difference in the rate of I_PIC_ activation and deactivation was the main cause of differences in the I–V (Figure 2(**F**)) and F–I curve (Figure 2(**H**) & (**I**)) between the anatomical and reduced model.

The second is the inability of the two-compartment model to represent the sparse distribution of PIC channels over the dendritic trees. This could limit the production of various types of PIC dynamics experimentally observed such as the staircase PICs [9]. The third one is that the application of the current two-compartment model could be further limited for the case where the distribution of synaptic inputs is not overlapped with that of PICs over a wide area of the dendrites. The second and third limitation of the current reduced modeling approach might be overcome by improving the cable models to incorporate the physiological properties of the asymmetric signal propagation in the dendrites.

The forth is related to the difference between the new and classic two-compartment modeling approach. Unlike the classic approach where individual model parameters are adjusted as a free parameter to satisfy the characteristic input-output relationship of the real cell [40], [55], all cable parameters (G_m,S_, G_m,D_, G_C_, C_m,S_ and C_m,D_) of the new approach used in the present study are simultaneously manipulated in a dependent manner to maintain the essential whole cell properties (R_N_ and τ_m_) and signal propagation properties (VA_SD_^DC^, VA_DS_^DC^ and VA_SD_^AC^) of the dendrites (see Eq. (4) – (8)). Consequently, the model parameter values of the new model may not be physiologically relevant (e.g. C_m_/G_m_ ratios at each compartment). However, our new approach can provide an analytical framework in which the model parameter values are uniquely determined to retain the biophysical properties that could be experimentally measured from dendritic neurons. In addition, the surface area (or p value in Eq. (5) – (8)) of each compartment in the new model is determined by the location of VGICs in the dendrites allowing for the simulation of varying VGICs in the dendrites based on the physical distance from the soma.

Lastly, the present study was focused on the interplay of plateau potential originated at the dendrites and nonlinear firing behavior of the cells. However, the detail of active conductances underlying the plateau potential was simplified using only L-type Ca channels as previously introduced [29], [33]. Thus, the maximum conductance (G_Ca,L_) of the L-type Ca channels may be underestimated in the present study due to the lack of inhibitory VGICs such as calcium dependent potassium currents. Further analysis would be needed for the situation where action potentials (e.g. Na or Ca spikes) are triggered locally at the dendrites, for neurons with somatic input resistances much higher than motoneurons, and for the morphological conditions that cause the similarity in the voltage attenuation properties in a wide variety of neurons [12].

### Implications to reduced modeling of dendritic neurons

The complex dendritic trees have been collapsed into the wide range of levels from multiple equivalent cables to one compartment in many reduced neuron models. The dendritic excitability in the reduced models depends on the reduction methods such as the conservation of surface area [56] or axial resistance [57]. However, it has not been shown to what degree the local excitability in the reduced dendrites is comparable with the anatomical case, resulting in the sacrifice of traceability between the anatomical and reduced models [58]. In the present study, we have shown the close relationship between voltage attenuation factors and dendritic excitability at all distances from the soma (Figure 3 and 6). This finding could provide a theoretical basis for physiologically representing the excitability of the dendrite in the reduced neuron models on the physical domain (i.e. D_path_), not on the electrical domain (i.e. length constant, λ) used up to date. Thereby the identification of voltage attenuation factors governing spatial heterogeneity of dendritic excitability may bridge the gap between the anatomically reconstructed and reduced neuron models.
